# Inhibition of CXCR2 alleviates the development of abdominal aortic aneurysm in Apo E^-/-^ mice

**DOI:** 10.1590/ACB360105

**Published:** 2021-02-15

**Authors:** Bo Sun, Fangda Li, Song Lai, Xu Zhang, Hongxia Wang, Yuan Li, Wei Wang, Yuexin Chen, Bao Liu, Yuehong Zheng

**Affiliations:** 1 Master. Chinese Academy of Medical Sciences – Peking Union Medical College Hospital – Department of Vascular Surgery – Beijing, China.; 2 Master. Weifang People’s Hospital – Department of Vascular Surgery – Weifang, Shandong, China.; 3 PhD. Weifang People’s Hospital – Department of Vascular Surgery – Weifang, Shandong, China.; 4 PhD. Ministry of Education – Peking University Third Hospital – Department of Cardiology and Institute of Vascular Medicine – Beijing Key Laboratory of Cardiovascular Receptors Research – Beijing, China.; 5 PhD. Capital Medical University – School of Basic Medical Sciences – Department of Physiology and Pathophysiology – Beijing, China.

**Keywords:** CXCR2, Angiotensin II, Apoptosis, Inflammation, Mice

## Abstract

**Purpose:**

To investigate the relationship between atherosclerotic abdominal aortic aneurysm (AAA) and CXC chemokine receptor type 2 (CXCR2).

**Methods:**

Mouse AAA model was established by embedding angiotensin-II pump (1000 ng/kg/min) in ApoE^-/-^ mice. Mice were received SB225002, a selective CXCR2 antagonist, for treatment. Blood pressure was recorded, and CXCR2^+^ macrophages were examined by flow cytometry analysis. Terminal-deoxynucleotidyl transferase mediated nick end labeling (TUNEL) staining was performed to detect cell apoptosis of abdominal aortic aneurysms. Macrophages were isolated from ApoE^-/-^ mice and treated with Ang II and/or SB225002. Dihydroethidium staining was carried out to determine reactive oxygen species (ROS) activity. Enzyme-linked immunosorbent assay (ELISA) was performed to determine the production of IL-1β and TNF-α. The corresponding gene expressions were measured using real-time polymerase chain reaction (PCR), western blot, and immunohistochemistry staining.

**Results:**

We found that Ang II activated the expression of CXCR2 in monocytes during the formation of AAA. Inhibition of CXCR2 significantly reduced the size of AAA, attenuated inflammation and phenotypic changes in blood vessels. Ang II-induced macrophages exhibited elevated ROS activity, and elevated levels of 1β and TNF-α, which were then partly abolished by SB225002.

**Conclusions:**

CXCR2 plays an important role in AAA, suggesting that inhibiting CXCR2 may be a new treatment for AAA.

## Introduction

Abdominal aortic aneurysm (AAA) refers to the irreversible dilatation of the renal inferior abdominal aorta, whose dilated diameter is ≥ 3.0 cm, and the risk factors of AAA include old age, male, smoking, obesity, etc. Particularly, AAA is one of the leading causes of death for people over 60 years old^1^^-^ 3. At present, the only treatment for AAA is open or endovascular surgical repair, and no pharmacological therapy has been admitted for clinical trials to limit or prevent the progressive expansion and rupture of AAA^4^. Therefore, the need for effective drug intervention made it meaningful to study the pathogenesis of AAA.

The pathologic manifestations of AAA include inflammatory reaction, matrix metalloproteinase (MMP) degradation, oxidative stress, angiogenesis, etc. Macrophages, as one of the important phagocytic and antigen-presenting cells in the body, play an important role in the inflammatory response during AAA progression^5^. Study on human AAA tissue showed that the number of inflammatory cells infiltrated by the outer membrane, mainly macrophages, increased with the gradual widening of AAA diameter^6^. These cells can directly or indirectly induce the proliferation, activation, differentiation, and apoptosis of other cells in the vascular wall by releasing proteolytic enzymes, such as cytokines and oxidation-derived free radicals, to regulate the remodeling of the vascular wall^7^.

CXC chemokine receptor type 2 (CXCR2), an important member of the CXC chemokine receptor family, is widely expressed in monocytes/macrophages, neutrophils, lymphocytes, fibroblasts, circulating endothelial progenitor cells, etc., playing an important role in the inflammatory processes^8^^,^ 9. C-X-C Motif Chemokine Ligand 1 (CXCL1)/CXCR2 biological axis plays an important role in the modulation of the adhesion and aggregation of macrophages in the aortic wall, and mediates the inflammatory response in vascular diseases such as hypertension and atherosclerosis^10^^,^ 11. In previous study, it was found that CXCR2 inhibitor (SB265610) alleviated the reduction of collagen deposition, elastin degradation, the metal matrix metalloprotease expression, and accumulation of macrophages in AAA^12^. However, there are still some limitations. Even though the protective role of CXCR2 inhibitor were demonstrated *in vivo*, *in vitro* study and the potential mechanism were still unclear. Therefore, in this article, the aim is to not only further verify the role of CXCR2 inhibition, but also explore its role *in vitro* and understand more about the potential mechanism of action.

## Methods

### Animal models

All animal experiments were approved by the Animal Care and Use Committee of Peking Union Medical College and conformed to the US National Institutes of Health Guide for the Care and Use of Laboratory Animal.

ApoE^−/−^ mice (male) were obtained from Vital River Laboratory Animal Technology Co. All mice were housed in a barrier facility, and ambient temperature ranged from 20–24 °C. Two batch of animal models were used in this research, and 5 mice were included in each group. The first batch: ApoE^−/−^ mice were subcutaneously infected with angiotensin II (Ang II; Sigma-Aldrich, St. Louis, MO) at a dose of 1000 ng/kg/min to induce AAA, or saline as control, using osmotic Mini-Pumps (Alzet MODEL 1007D; DURECT, Cupertino, CA) for 28 days at 10 weeks of age. The second batch: ApoE^−/−^ mice were divided into four groups: saline, SB225002, Ang II, and Ang II+SB225002. SB225002, N-(2-hydroxy-4-nitrophenyl)-N’-(2-bromophenyl) urea, is a selective CXCR2 antagonist (Calbiochem, San Diego, CA, USA). Mice were treated intraperitoneally with 10 mg/kg of SB225002 with or without Ang II infusion.

### Blood pressure recordings

Blood pressure was measured by the tail-cuff system (SoftronBP-98A; Softron, Tokyo, Japan) and the telemetric blood pressure system (TA-PA11C10, Data Science International, Tilburg, The Netherlands).

### Analysis of AAA

Abdominal aortic aneurysm diameter was measured with a Vevo 770 ultrasound system (VisualSonics Inc.) according to the protocol of the manufacturer.

### Real-time Polymerase Chain Reaction (PCR)

Total RNA was extracted using TRIzol Reagent (Invitrogen Corp) and reversely transcribed into cDNA by using Transcriptor First Strand cDNA Synthesis Kit (Roche, Germany). Real-time PCR was performed by SYBR PrimeScript RT-PCR Kit II (TaKaRa, Japan). The oligonucleotide primers were listed as follows: CXCR2 Forward Primer: 5’-ATG CCC TCT ATT CTG CCA GAT -3’; Reverse Primer: 5’-GTG CTC CGG TTG TAT AAG ATG AC-3’. OPN Forward Primer: 5’-CAC TCC AAT CGT CCC TAC AGT-3’; Reverse Primer: 5’-CTG GAA ACT CCT AGA CTT TGA CC-3’. α-SMA Forward Primer: 5’-GGC ATC CAC GAA ACC ACC TA -3’; Reverse Primer: 5’-TTC CTG ACC ACT AGA GG GGG-3’. TNF-α Forward Primer: 5’-TAG CCC ACG TCG TAG CAA AC-3’; Reverse Primer: 5’-ACC CTG AGC CAT AAT CCC CT-3’; IL-1β forward primer: 5’-CAA CCA ACA AGT GAT ATT CTC CAT G-3’; reverse primer: 5’-GAT CCA CAC TCT CCA GCT GC A -3’; glyceraldehyde-3-phosphate dehydrogenase (GAPDH) forward primer: 5’-GGT TGT CTC CTG CGA CTT CA-3’; reverse primer: 5’-GGT GGT CCA GGG TTT CTT ACT C-3’. The 2^-△△CT^ method was used to determine fold changes. GAPDH was used in each sample as a house-keeping gene to standardize the results by eliminating variations in mRNA quantity.

### Western blot analysis

The abdominal aortic aneurysm tissues were lysed in extraction buffer containing: Tris/HCl 50 mM (pH 7.4), KCl 154 mM, glucose 5 mM, EDTA 0.5 mM, PMSF1 mM, DTT 2 mM, and 1% Triton X-100. Proteins were separated by sodium dodecyl sulfate polyacrylamide gel electrophoresis (SDS-PAGE), and transferred to polyvinylidene fluoride membranes (Millipore). The membranes were blocked in Tris-buffered saline containing Tween-20 (TBST) for 2 h, followed by an overnight incubation at 4 °C with primary antibodies against CXCR2 (1:1000; Abcam), α-SMA (1: 1000; Abcam), TGF-β1 (1: 1000; Abcam), OPN (1: 1000; Abcam), and GAPDH (1:2000; polyclonal antibody; Sigma Chemical Co.). After being washed by TBST, the membranes were incubated with HRP-conjugated secondary antibody (1:2000) at room temperature for 2 h. The internal control was GAPDH.

### Immunohistochemistry (IHC) staining

Immunohistochemistry (IHC) staining was performed with primary antibody CXCR2. Digital photographs were taken at 200× magnification of over 10 random fields from each aorta. The images were captured using a Nikon Labophot 2 microscope (Nikon, Tokyo, Japan).

### Flow cytometry

Flow cytometry was performed as mentioned earlier^12^. Blood vessels were perfused with phosphate buffer (PBS) to clear blood cells. After dissection, the blood vessels were cut up, and then digested with 0.125% trypsin, 0.1% type I collagenase, 0.1% type II collagenase, and 0.1% type IV collagenase in PBS at 37 °C for 1 h, the cell suspension was centrifuged at 300× g at 4 °C for 5 min, and the supernatant was passed through the 70-μL filter membrane. The isolated cells were stained with antibodies and their homologous matched negative controls were sorted by FACS Fortessa FLOW cytometer.

### TUNEL staining for tissues

According to the instructions, apoptosis was detected by TUNEL staining (Red, TUNEL kit; Roche Germany). The samples were fixed with 4% paraformaldehyde, dehydrated and embedded tissue sections (5 mm) with paraffin. After30 min, they were treated with protease K at 37 °C, and the slices were washed with PBST for 3 times, with 5 min each time. The samples were incubated with terminal deoxynucleotidyl transferase (TDT) and biotin-16-dUTP in the mixture of TDT buffer reaction at 37 °C for 1 h. After washing with PBST for 3 times, the nuclei were stained with diamino-2-phenylindole (DAPI). The sections were then observed under a fluorescence microscope (200×) (Olympus BX53; Olympus, Tokyo, Japan). An average of 5 fields were observed in each specimen. The apoptosis index of these areas was calculated as the percentage of positive cells.

### Cell culture and treatment

Mouse peritoneal macrophages were isolated according to a previous study^13^. After mice were euthanized, the outer layer of the peritoneum was incised using scissors, and 3% thioglycolate medium (2.5 mL) was injected into the peritoneal cavity. Then, the peritoneal cells were exuded from peritoneal cavities, washed and resuspended in DMEM/high glucose media (Hyclone, USA) with 10% fetal bovine serum (Gibco LifeTechnologies) and 1% (v/v) penicillin. Cells were then cultured in 6-well plates at a density of 1 × 10^6^ per well at 37 °C. The nonadherent cells were gently removed 2 h after incubation, and the left pure macrophages were collected and cultured in 6-well plates. The primary culture of mouse peritoneal macrophages was then stimulated by Ang II and treated with SB225002 for further experiments.

### Dihydroethidium (DHE) staining

The mouse peritoneal macrophages were stimulated by Ang II and/or treated with SB225002 for 24 h. Then, the level of active oxygen metabolism was detected using dihydroethidium.

### Enzyme-linked immunosorbent assay (ELISA)

The cell culture medium supernatants were collected. The concentrations of IL-1β and TNF-α were determined using their corresponding ELISA kits (R&D Systems) according to the manufacturer’s instructions.

### Statistical analysis

The statistical analyses were performed by using GraphPad (8.0.1). All the results were generated from three independent experiments. Differences in continuous variables between groups were determined by Student’s t-test or one-way analysis of variance followed by Tukey’s post hoc analysis. P < 0.05 was considered statistically significant for all analyses.

## Results

### Ang II induced AAA formation in ApoE^−/−^ mice

**Figure 1:**
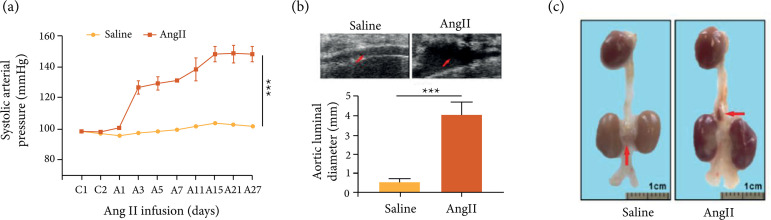
Ang II induced AAA formation in ApoE−/− mice. (a) The noninvasive tail-cuff method was used to measure the systolic blood pressure of mice in each group; (b) Representative images were selected to show high-frequency ultrasonography of abdominal aorta; (c) Representative aortas of Saline and Ang II group. C1: The first day before Ang II induction; A1: The first day after Ang II induction. *P < 0.001.

To verify the successful establishment of the AAA model, the blood pressure of mice treated with or without Ang II was measured. It was found that the blood pressure of mice treated with Ang II increased significantly (Fig. 1a). Next, the results of ultrasonic examination of abdominal aortas showed that the size of abdominal aortas was significantly larger by high-frequency ultrasound (Fig. 1b). Besides, Ang II could increase aneurysm formation (Fig. 1c).

### CXCR2+ macrophages were up-regulated in abdominal aortic aneurysm animal model

**Figure 2:**
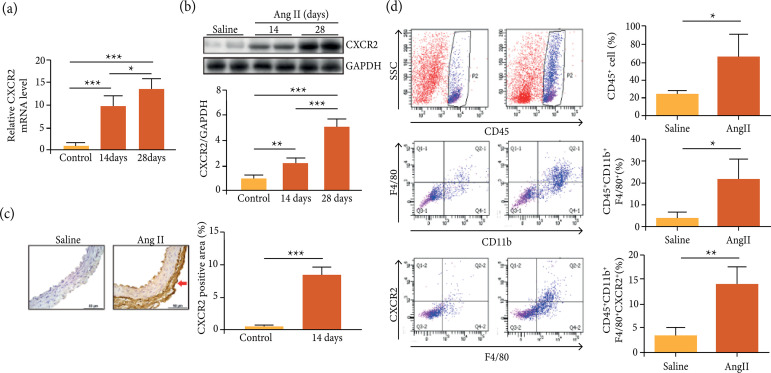
CXCR2+macrophages were up-regulated in abdominal aortic aneurysm animal model. (a-b) The expression of CXCR2 was examined by real time PCR and western blotting in the aortas of mice infused with Ang II for 14 and 28 days; (c) The expression of CXCR2 was examined by IHC staining in the aortas of mice infused with Ang II for 14 days; (d) The representation of quantifying the number of CD45+ neutrophils, CD45+CD11b+F4/80+ macrophages and CD45+CD11b+F4/80+CXCR2+ macrophages in the aortas of mice by flow cytometry analysis; *p < 0.05, **p < 0.01, ***p < 0.001.

To investigate the role of CXCR2 in AAA animal model, the expression level of CXCR2 was examined. The results showed that the mRNA and protein levels of CXCR2 were obviously increased after the vessels were infused with Ang II (Fig. 2a-b).CXCR2 was markedly up-regulated in Ang II-infused vessels compared with control by IHC staining (Fig. 2c). Flow cytometry showed that Ang II infusion caused increase of CD45+CXCR2+cells, including CD11b+CXCR2+ monocytes and CD11b+F4/80+CXCR2+ macrophages in the AAA (Fig. 2d).

### Inhibition of CXCR2 suppressed Ang II-induced hypertension and severity of abdominal aortic aneurysms in ApoE^-/-^ mice

**Figure 3:**
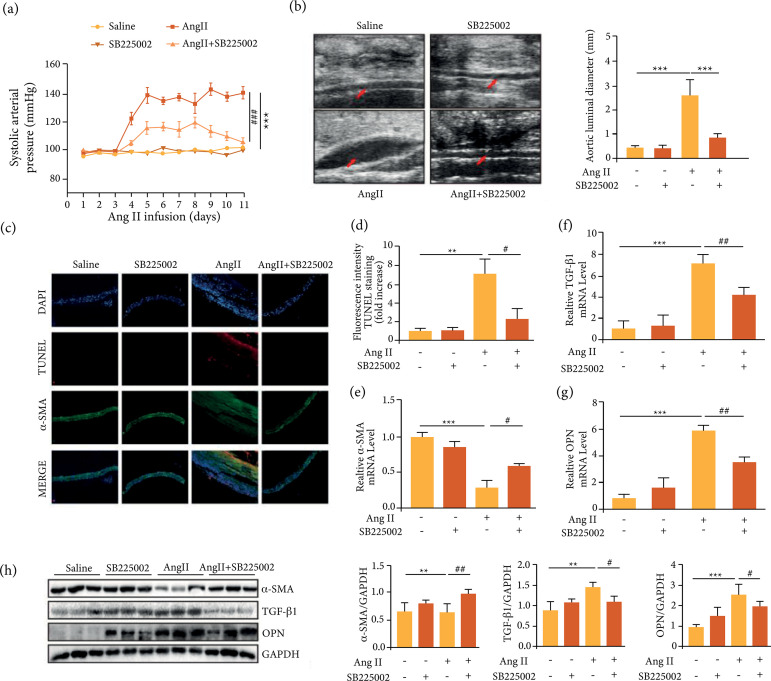
Inhibition of CXCR2 suppressed Ang II-induced hypertension, the incidence and severity of abdominal aortic aneurysms and inflammation in ApoE-/- mice. (a) The noninvasive tail-cuff method was used to measure the systolic blood pressure of mice in each group; (b) Representative aortas in ApoE-/- mice induced by Ang II with or without SB225002 treatment; (c-d) TUNEL staining of the abdominal aortic aneurysms and the TUNEL fluorescence (red) intensity of each group were analyzed; (e-g) qPCR analysis of TGF-β1, α-SMA, and OPN; (h) Western blotting analysis of α-SMA, TGF-β1 and OPN; */#p < 0.05, **/##p < 0.01, ***/###p < 0.001.

Next, the influence of CXCR2 on blood pressure in Ang II-induced ApoE^-/-^mice was examined using the noninvasive tail-cuff method. The results showed that the blood pressure was significantly increased upon Ang II induction, which was then obviously alleviated upon cotreatment with SB225002, indicating an obvious remission of CXCR2 inhibition on high blood pressure (Fig. 3a). Besides, inhibition of CXCR2 significantly inhibited the AAA size induced by Ang II, which was confirmed by high-frequency ultrasound (Fig. 3b). TUNEL assay was then used to verify the effect of CXCR2 on vascular tissue apoptosis, and the results showed that inhibition of CXCR2 inhibited Ang II-induced apoptosis (Fig. 3c-d). In addition, Ang II decreased the expression of α-SMA and increased the expression of OPN and TGF-β1 in ApoE^-/-^ mice, while inhibition of CXCR2 improved the expression of α-SMA and inhibited the expression of TGF-β1 and OPN (Fig. 3e-h).

*Inhibition of CXCR2 suppressed reactive oxygen species (ROS) production and inflammatory responses induced by Ang II in macrophages*

**Figure 4:**
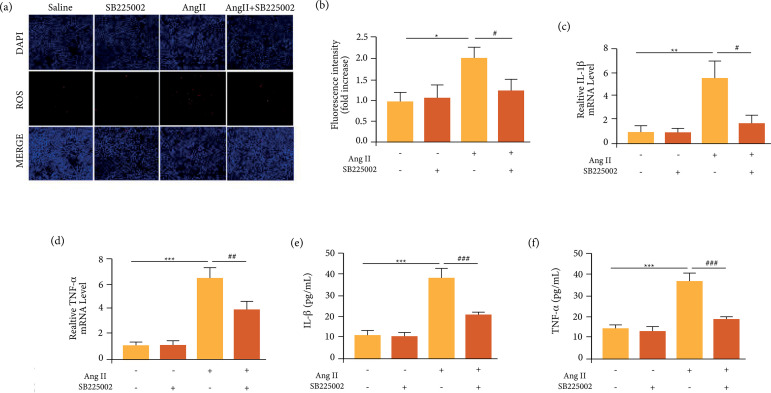
Inhibition of CXCR2 suppressed Ang II-induced ROS production and inflammatory responses in vitro. (a-b) DHE staining of the macrophages and the ROS fluorescence intensity of each group were analyzed; (c-d) qPCR analysis of IL-1β and TNF-α. (e-f) ELISA analysis of IL-1β and TNF-α; */#p < 0.05, **/##p < 0.01, ***/###p < 0.001.

Then, peritoneal macrophages from ApoE^-/-^ mice were extracted and cultured, and subsequently received Ang II and/or SB225002, similar as the AAA model *in vivo*. It was found that Ang II significantly induced reactive oxygen species (ROS) production. SB225002 alone had no effect on ROS activity, but could significantly decrease the elevated ROS activity induced by Ang II (Fig. 4a-b). Besides, Ang II also significantly increased the mRNA level of IL-1β and TNF-α, which was then reversed by SB225002 (Fig. 4c-d). The similar changes of IL-1β and TNF-α were also found by ELISA assays (Fig. 4e-f). Thus, inhibition of CXCR2 could attenuate ROS activity and inflammatory responses induced by Ang II.

## Discussion

It is well known that abdominal aortic aneurysm is a fatal cardiovascular disease characterized by the dilation of the abdominal aorta. It is caused by the accumulation of immune cells, inflammation and medial degeneration, which in turn leads to rupture and bleeding of the aorta^14^. Currently, the standard of care for AAA is limited to surgery at a later stage of disease progression. Therefore, it is needed to better understand the mechanism, especially the inflammatory property of AAA. Various risk factors for AAA have been studied, including elevated blood pressure mediated by activation of the renin-angiotensin system (RAS) and upregulation of Ang II^15^. Currently, Ang II-induced AAA model has been widely recognized, which recapitulates many aspects of human AAA, such as elevated blood pressure, extracellular matrix degradation, and the gradual recruitment of immune cells^16^. Thus, Ang II-induced AAA model is used for our research.

It has been proved that a variety of cytokines can stimulate leukocyte migration in the process of inflammation^17^. Chemokines belong to the family of cytokines, which can induce neutrophils and monocytes/macrophages to migrate to the damaged blood vessel wall. In mice, chemokine can recruit neutrophils and monocytes through chemokine receptor CXCR2. Increasing evidence shows that CXCR2 plays a key role in oncology, cardiovascular development, and various inflammatory diseases^18^^,^ 19. In this paper, the relationship between AAA and CXCR2 was further studied. A mouse abdominal aortic aneurysm model was established by embedding an Ang II pump (1000 ng/kg/min) in ApoE^-/-^ mice. Ang II activated monocytes to express CXCR2 (CD45^+^CD11^+^bF4/80^+^) during AAA formation. At the same time, it was found that inhibition of CXCR2 could significantly reduce the size of AAA and attenuate inflammation and vascular phenotypic changes. Inhibition of CXCR2 could attenuate ROS activity and inflammatory responses induced by Ang II in macrophages, which indicated that CXCR2 played an important role in inflammation and oxidative stress during AAA. Thus, suppressing the expression of CXCR2 may be a new method for the treatment of AAA.

During the progression of AAA, the homeostasis of vascular smooth muscle cells (VSMCs) is disturbed, and the transformation of VSMCs from contractile phenotype to secretory phenotype plays an important role in vascular pathology, thus contributing to AAA formation^20^. Alpha-SMA and OPN can be used as marker proteins to identify the phenotype of vascular smooth muscle cells. A study has found that the expression of OPN in human abdominal aortic aneurysm increased more than 125 times^21^. In this paper, it was revealed that in ApoE^-/-^ mice, Ang II decreased the expression of α-SMA and increased the expression of OPN and TGF-β1, while inhibition of CXCR2 improved the expression of α-SMA and inhibited the expression of TGF-β1 and OPN in Ang II-treated ApoE^-/-^ mice, indicating that inhibition of CXCR2 might prevent AAA by inhibiting VSMCs phenotypic switching.

## Conclusions

This study showed that inhibition of CXCR2 prevented aortic wall destruction partly through inhibiting VSMCs phenotypic switching, decreasing cell apoptosis and inflammatory response, thus suppressing AAA formation. This finding provided more theoretical basis for AAA pathomechanism and treatment.
